# Transcrystallization of Isotactic Polypropylene/Bacterial Cellulose Hamburger Composite

**DOI:** 10.3390/polym11030508

**Published:** 2019-03-18

**Authors:** Bo Wang, Fu-hua Lin, Xiang-yang Li, Xu-ran Ji, Si-xiao Liu, Xiao-jing Han, Zheng-qiu Yuan, Jun Luo

**Affiliations:** 1 School of Chemistry and Biological Engineering, Taiyuan University of Science and Technology, Taiyuan 030024, China; 13546474299@163.com (B.W.); jxr12345@stu.tyust.edu.cn (X.-r.J.); liusixiao@stu.tyust.edu.cn (S.-x.L.); 201821020235@stu.tyust.edu.cn (X.-j.H.); 2 Key Laboratory of Renewable Energy, Guangzhou Institute of Energy Conversion, Chinese Academy of Sciences, Guangzhou 510640, China; 3 Shanxi Provincial Institute of Chemical Industry, Taiyuan 030021, China; kelin0514@163.com (F.-h.L.); 17835611229@163.com (X.-y.L.); 4 School of Chemistry and Chemical Engineering, Hunan University of Science and Technology, Xiangtan 411201, China; yuanzhengqiu@126.com; 5 Guangzhou Fibre Product Testing and Research Institute, Guangzhou 510220, China

**Keywords:** isotactic polypropylene, bacterial cellulose, hamburger composite, transcrystallization

## Abstract

Isotactic polypropylene (iPP) is a commonly used thermoplastic polymer with many excellent properties. But high brittleness, especially at low temperatures, limits the use of iPP. The presence of transcrystallization of iPP makes it possible for fiber-reinforced iPP composites with higher strength. Bacterial cellulose (BC) is a kind of cellulose with great potential to be used as a new filler to reinforce iPP due to its high crystallinity, biodegradability and efficient mechanical properties. In this study, the iPP/BC hamburger composite was prepared by a simple hot press and maleic anhydride grafted polypropylene (MAPP) was used to improve the interface compatibility of iPP and BC. The polarizing microscope (POM) photograph shows that BC successfully induces the transcrystallization of iPP. The differential Scanning Calorimeter (DSC) date proves that the addition of BC could improve the thermal properties and crystallization rate of the composite. Especially, this change is more obvious of the iPP/MAPP/BC. The mechanical properties of the iPP/BC composites were greatly increased. This DSC date is higher than BC; we used BC particles to enhance the iPP in our previous research. The scanning Electron Microscope (SEM) analysis intuitively shows that the interface of the iPP/MAPP/BC is more smooth and flat than the iPP/BC. The fourier Transform infrared spectroscopy (FT-IR) analysis of the iPP/BC hamburger composites was shown that a new C=O group vibration appeared at 1743 cm^−1^, which indicated that the hydrogen bond structure of BC molecules was weakened and some hydroxyl groups were substituted after modification which can increase the lipophilicity of BC. These results indicated that the BC fiber can easily induce the transcrystallization of iPP, which has excellent mechanical properties. Moreover, the addition of MAPP contributes greatly to the interface compatibility of iPP and BC.

## 1. Introduction

Isotactic polypropylene (iPP) is a kind of semi-crystallization thermoplastic polymer with many excellent properties such as excellent heat resistance, chemical stability, high dielectric coefficient and mechanical properties [1,2,3]. Unfortunately, high brittleness limits the use of iPP especially at low temperatures [4]. At present, iPP has generally reinforced with various types of organic or inorganic fillers [5,6,7]. Among them, fiber-reinforced iPP composites have been widely used in automobile, agriculture and civil engineering due to their excellent properties including their light weight, low price and high strength [8]. From the crystallographic point of view, the most common crystal form of iPP is spherulite. The presence of the transcrystallization of iPP makes it possible for fiber-reinforced iPP composites with higher strength, which has attracted a good deal of attention [9].

In general, the fibers were embedded in the iPP matrix and the transcrystallization is induced by the preferential nucleation of crystals at the fiber surface [10]. As the iPP melt is allowed to cool when in contact with fibers—a source of nucleating centers—the proximity of these sites on the surface inhibits lateral growth of the resultant spherulites, and as a result the crystallization develops only in a direction normal to the fiber surface [11]. Because the addition of fibers is equivalent to introducing nuclei into iPP matrix, it has a heterogeneous nucleation effect. In this case, the crystal growth changes from the radial direction of spherulites to the direction normal to the fiber surface as nucleation occurs with a sufficiently high density along the fiber surface results in a columnar transcrystallization layer [12].

Cellulose fiber is a high crystallization natural polymer with abundant nucleation sites and was widely used in reinforced iPP. In recent years, cellulose fiber-reinforced iPP made great breakthroughs and has been widely used in many fields [13]. Gray [14] used prepared cellulose nanocrystal from cotton, treated it with sulfuric acid, and first observed the transcrystallization of iPP along the cellulose I surface. This interesting result provided a new idea for the study of the transcrystallization of iPP.

Bacterial cellulose (BC) is a kind of cellulose commonly used in food, specialty paper, speaker membranes and biomedical applications [15,16,17]. Compared with cellulose, BC has many excellent properties such as a high degree of crystallinity, high purity and high mechanical properties (Young’s modulus of BC fiber is as high as 114 GPa). Especially, the BC is a network of ultrafine fibers with a diameter of 0.01–0.1μm. Each filamentous fiber is composed of a certain number of microfibers. The adjacent microfibers are transversely connected by hydrogen bonds to form microfibers with a diameter of about 3–4 nm. Multiple microfibers are combined to form febrile ribbons of variable length, 30–100 nm in width and 3–8 nm in thickness [18]. Therefore, the special three-dimensional network structure can better serve as heterogeneous nucleation point to induce crystallization of iPP and has great potential for reinforcing iPP. But nonpolarity iPP and polar BC are usually incompatible, which leads to the weak iPP/BC interfacial adhesion thus limits the mechanical properties of the composites. From the point of view of polymer crystallization, if the interface compatibility between iPP and BC is poor, the interface crystallization and bonding will not be stable. On the contrary, if the interface compatibility is good, compact microcrystals or cross-crystallization will be formed at the interface between the iPP and BC, thus greatly improving the mechanical properties of the iPP.

Maleic anhydride grafted polypropylene (MAPP) as a compatibilizer has been used to improve interfacial compatibility of iPP and BC with good results. Because the molecular chains of MAPP contain carboxyl and iPP chains. On the one hand, the carboxyl can esterify with hydroxyl groups on the surface of BC, and then graft onto the surface of BC to reduce its polarity. On the other hand, the iPP chains in MAPP can form cocrystallization with the matrix of iPP. In our previous research, renewable BC reinforced iPP composites already show promising mechanical properties [19]. Furthermore, it’s interesting to know whether BC induced iPP can also produce transcrystallization.

In this study, iPP/BC hamburger composite was successfully prepared by a simple hot press. A POM was used to observe the crystal morphology of iPP/BC composites. The effects of BC and MAPP addition on the crystallization behavior, mechanical properties changes of the composites were investigated. Meanwhile, the FT-IR and SEM measurements were used for compatibility analysis of iPP/BC composites.

## 2. Materials and Methods

### 2.1. Materials

iPP (S1003) film (100 mm × 10 mm × 0.5 mm), MW = 3.2 × 105 g/mol, and the melt flow index (MFI) of 3.6 g/10 min was purchased from Sinopec Beijing Yanshan Company (Beijing, China) The BC film was obtained from Hainan Yida Food Industry Co., Ltd. (Hainan, China). It was flushed repeatedly by deionized water until neutral, freeze-dried and then cut into strips (100 mm × 1 mm). MAPP film (100 mm × 5 mm; 1% grafting) was supplied by Nantong Sunny Polymer New Material Technology Co., Ltd. (Nantong, China).

### 2.2. Preparation of iPP/BC Hamburger Composites

**Figure 1:**
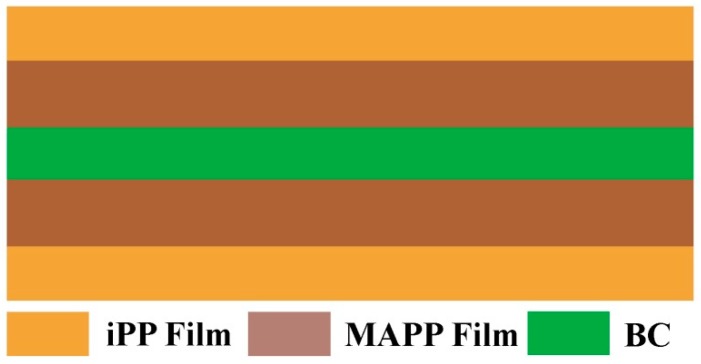
Schematic diagram of iPP/BC hamburger composites.

The iPP film was evenly placed in a mold on a press vulcanizer (XH-406, Dongguan Xihua Testing Instrument Co., Ltd., Dongguan, China) at 200 °C. Next, a BC strip was overlaid on the iPP film, and then another iPP film has overlaid the BC like a “hamburger” (Figure 1). Finally, the sample was hot pressed (10 MPa) into slice. Similarly, the BC strip was overlaid between two MAPP films first, and then this “hamburger” was introduced into two pieces of iPP film.

### 2.3. Characterization of iPP/BC Composites

#### 2.3.1. Polarizing Microscope (POM)

The POM observations were performed on the composites with a Leica microscope (DFC295, Leica Microsystems Corporation, Wetzlar, Germany) with a temperature controlled stage (LTS 420, Linkam Scientific Instruments, Tadworth, UK). The sample was first heating to 220 °C and maintained for 3 min, and then cooling down to 138 °C with the cooling rate of 50 °C/min maintained for 30 min. The crystal morphology of the sample can be observed at a constant temperature.

#### 2.3.2. Differential Scanning Calorimeter (DSC) Testing

The DSC testing of the iPP/BC hamburger composites was performed on a DSC (DSC1, Mettler Toledo Corporation, Greifensee, Switzerland). Samples were firstly heated to 220 °C, maintained at 220 °C for 3 min, and then cooled using the cooling rate of −80 °C/min to 138 °C, which was maintained for 400 min. The isothermal crystallization time was set as 400 min. The sample was heated using heating rate 10 °C/min to 220 °C. The isothermal crystallization and reheating curves were used for analysis.

#### 2.3.3. Mechanical Properties

Tensile tests were performed using a universal testing machine (Legend 2367, Instron Company, Norwood, MA, USA) with a speed of 10 mm/min according to GB/T 1040.3-2006. The impact strength of the composites measured using a plastic film pendulum impact tester (FIT-01, Jinan Languang Mechanical and Electrical Technology Co., Ltd., Jinan, China) with 1J capacity at the maximum pendulum angle (120°) according to GB/T 8809-2015. Ten replicates of each sample were tested with a mechanical test.

#### 2.3.4. Scanning Electron Microscope (SEM)

The interface between the BC fiber and iPP of the composites was characterized by a scanning electron microscope (JSM-IT200, JEOL, Tokyo, Japan) at an accelerated voltage of 2 kV.

#### 2.3.5. FT-IR Analysis

Fourier transform infrared (FT-IR) was performed on an FT-IR spectrometer with an ATR accessory (Nicolet iS50, Thermo Scientific Inc., Waltham, MA, USA) taking 64 scans for each sample.

## 3. Results and Discussion

### 3.1. Transcrystallization of the iPP/BC Hamburger Composites

**Figure 2:**
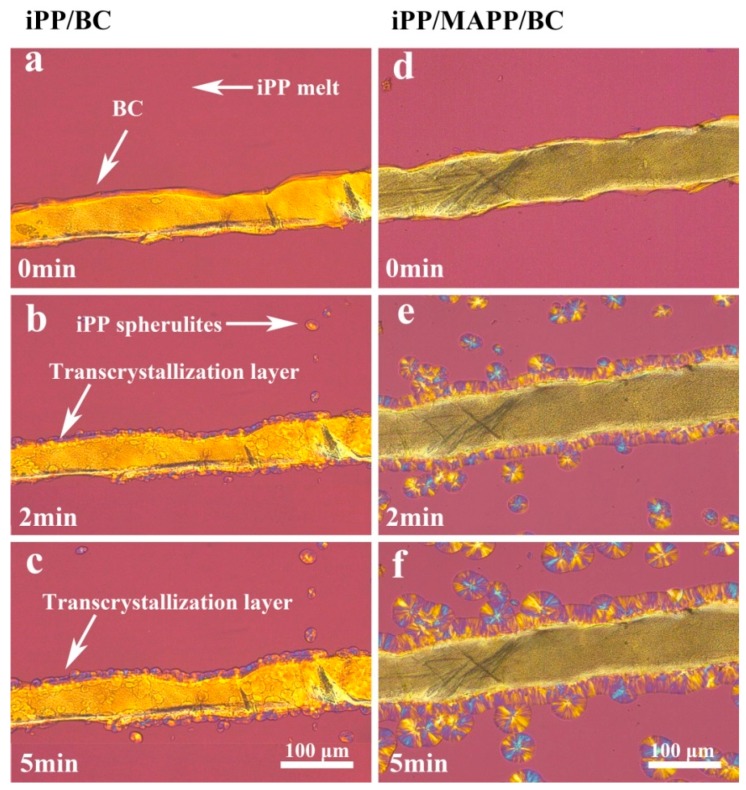
POM photograph showing transcrystallization of iPP/BC hamburger composite at 138 °C ((**a**–**c**) iPP/BC; (**d**–**f**) iPP/MAPP/BC).

The BC particle can increase the crystallization rate of iPP because of the nucleation effect of the BC in the iPP. This viewpoint has proved in our previous research [20]. So how does BC fiber affect the crystallization of iPP? The POM photograph of iPP/BC composites at 138 °C is shown in Figure 2. In the early stage of crystallization, it can only see the BC fiber and the iPP crystal does not appear (Figure 2a,d). After 2 min, the iPP crystal begins to grow. It can be seen that the amount of the crystal and the crystal size of the iPP/MAPP/BC is bigger than the iPP/BC (Figure 2b,c,e,f). This proved that the crystallization rate of the iPP/MAPP/BC is higher than the iPP/BC composites. This is also evidence of the compatibility improvement of the iPP/MAPP/BC composites. Delighting, the transcrystallization layer is found in the POM photograph. It is proved that the crystallization firstly starts at the interfaces of the BC, then extends to the iPP layers and eventually forms the transcrystals [21]. Even in the early stages of the crystallization, the nucleation along the edge of the BC film is very dense, so that the edge is completely covered by a transcrystallization layer, far from the extensive nucleation in the bulk. Moreover, while the BC fiber edge enhances nucleation, the growth rate of the transcrystallization layer is approximately the same as that as the growth of the bulk spherulites, where the width of the layer is approximately equal to the radius of the bulk spherulites [22]. Furthermore, with the increase of crystallization time, the thickness of transcrystallization layer increases, the transcrystallization layer of iPP/BC is thicker than the iPP/MAPP/BC. It indicates that the addition of MAPP improves the compatibility between iPP and BC.

### 3.2. DSC Testing of the iPP/BC Hamburger Composites

**Figure 3:**
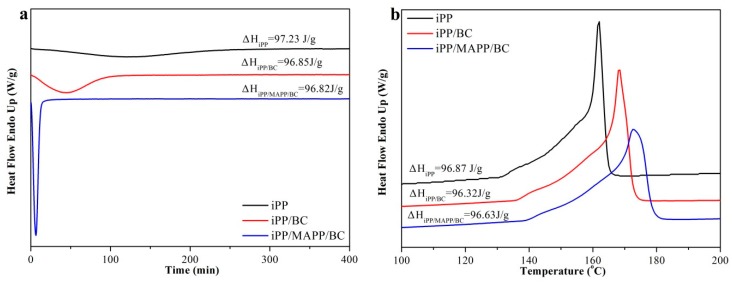
DSC curves of the iPP/BC hamburger composite (**a**) isothermal crystallization curve; (**b**) reheating curve).

The DSC curves of the iPP/BC hamburger composite were shown in Figure 3 (The direction indicated by the arrow is endothermic). It can be seen from the isothermal crystallization curves (Figure 3a) the crystallization peak becomes narrower which indicated the crystallization time of the composite decreased. In addition, the area of the crystallization or melting peak represents the enthalpy of endothermic or exothermic of the sample. The date of crystallization enthalpy and the fusion enthalpy (ΔH) of the sample was scarcely less which indicate that the crystallization time is shorter (the crystallization peak becomes narrower), the area of the crystallization peak will remain unchanged, and the crystallization peak will become deeper. This phenomenon proved that BC fiber can greatly accelerate the crystallization rate of iPP [23]. Meanwhile, the reheating curves of the composite (Figure 3b) show that the melting temperature of the composite is shifted to a higher temperature value. The result suggested that the addition of BC could improve the thermal properties of the composite which making the composite can be processed at high temperatures and greatly shortening the processing cycle. Especially, the crystallization rate and the melting temperature of the iPP/MAPP/BC are higher than iPP/BC, which indicated that the compatibility of the iPP and BC has a great influence on the crystallization behavior of the composite; the addition of the MAPP improved the compatibility of the iPP and BC.

### 3.3. Mechanical Properties of the iPP/BC Hamburger Composites

**Figure 4:**
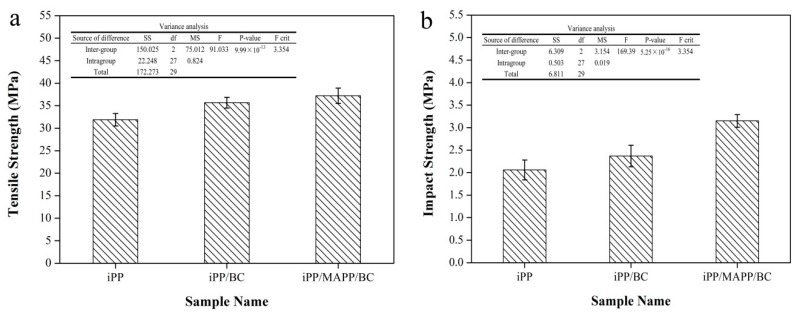
Mechanical properties of the iPP/BC hamburger composite: (**a**) tensile strength; (**b**) impact strength).

As we know, the change of the crystal structure of transcrystallization polymers will lead to the change of mechanical properties of the polymers. In our previous research, we used BC particles to enhance the iPP and the impact strength of the iPP increased by 27% and the BC particles did not change the crystal structure of the iPP [20,24]. The POM photograph shows that the transcrystallization has appeared in the hamburger composite. How does the transcrystallization affect the mechanical properties of the composite? The tensile strength and impact strength of the iPP/BC hamburger composites is given in Figure 4. The tensile strength of the composites was greatly increased from 31.89 to 35.68 and 37.21 MPa, respectively, representing 11.88% and 16.68% increase over pure iPP, and the impact strength of the composites was increased from 2.06 to 2.37 and 3.15 KJ/m^2^, respectively, representing 15.05% and 52.91% increase over pure iPP. This date is higher than our previous research. It proved that the appearance of the transcrystallization greatly improved the mechanical properties of the iPP/BC hamburger composite. Meanwhile, the variance analysis of the mechanical data in the Figure 2 shows that F > F_0.01_(2,27) = 3.354, *P*-value < 0.01 (tensile strength), F > F_0.01_(2,27) = 3.354, *P*-value < 0.01 (impact strength). The variance analysis data show that the BC and MAPP has a very significant influence on the tensile properties of iPP. Moreover, the addition of MAPP can significantly improve the compatibility between components in the composites, which then show better mechanical properties.

### 3.4. SEM Photographs on the Interface of the iPP/BC Hamburger Composite

**Figure 5:**
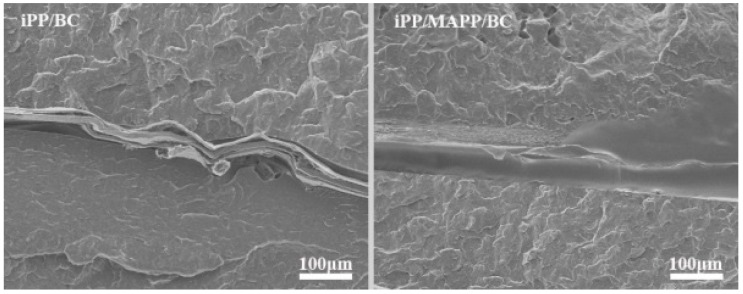
SEM photographs on the interface of the iPP/BC hamburger composite.

In order to further study the interface compatibility between iPP and BC, the SEM photographs on the interface of the iPP/BC hamburger composite was shown in Figure 5. It can be seen intuitively from Figure 5 that the structure of the composite is the hamburger-like, composed of BC encapsulated by iPP matrix on both sides. There are many voids, convex and folds on the interface of the iPP/BC which can easily form cracks and defects and fracture when the composite subjected to external forces. On the contrary, the interface of the iPP/MAPP/BC is smooth and flat which shows better compatibility. The SEM results indicated that the compatibility between iPP and BC getting better by added MAPP which can greatly improve the mechanical properties of the iPP/BC hamburger composite.

### 3.5. FT-IR of the iPP/BC Hamburger Composite

**Figure 6:**
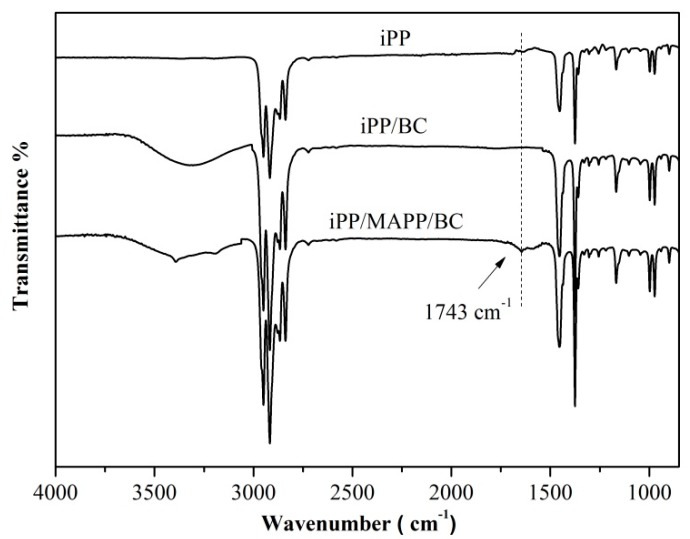
FTIR of the iPP/BC hamburger composite.

The FT-IR spectra of iPP and iPP/BC hamburger composites were illustrated in Figure 6. The O-H stretching vibration absorption peak was shown in the FT-IR spectra of iPP/BC hamburger composites. Especially, a new C=O group vibration appeared at 1743 cm^−1^ for sample iPP/BC and iPP/MAPP/BC [25]. The effect of the addition of MAPP was confirmed by this absorption peak. Besides, compared to iPP/BC, the O–H stretching vibration absorption peak of iPP/MAPP/BC moved to a higher wavenumber (3330–3386 cm^−1^), peak intensity weakened, and the width of the peak became narrowed, indicating that the hydrogen bond structure of BC molecules was weakened and some hydroxyl groups were substituted after modification, which can increase the lipophilicity of BC. The good compatibility between iPP and BC was obtained.

## 4. Conclusions

In this work, the iPP/BC hamburger composite was prepared by a simple hot press (the BC were embedded in the iPP matrix). MAPP was used to improve the interface compatibility of iPP and BC. The POM photograph shows that BC successfully induces the transcrystallization of iPP. The iPP/BC composite crystallizes first at the interface and then extends to the iPP layer and eventually forms a transcrystallization layer. Furthermore, compared to iPP/BC composites, the spherulite size is denser in the iPP matrix of iPP/MAPP/BC composites. The DSC date proves that the addition of BC could improve the thermal properties and crystallization rate of the composite. Especially, this change is more obvious of the iPP/MAPP/BC. Compared to the neat iPP, the tensile strength and impact strength of the iPP/BC composites were greatly increased. This DSC date is higher than BC; we used BC particles to enhance the iPP in our previous research. The SEM analysis intuitively shows that the interface of the iPP/MAPP/BC is more smooth and flat than the iPP/BC. The FT-IR analysis of iPP/BC composites was shown that a new C=O group vibration appeared at 1743 cm^−1^, which indicated that the hydrogen bond structure of BC molecules was weakened and some hydroxyl groups were substituted after modification—this can increase the lipophilicity of BC. In summary, this work indicated that the BC fiber can easily induce the transcrystallization of iPP, which has excellent mechanical properties. Moreover, the addition of MAPP contributes greatly to interface compatibility of iPP and BC.
